# Identification of Slime Mold Metabolites That Confer Protection to Commercial Crops against Root-Knot Nematodes

**DOI:** 10.1021/acs.jafc.5c04345

**Published:** 2025-07-29

**Authors:** Kana Y. Hayashi, Yukiko Nagamatsu, Moemi Kawano, Sayaka Fuchimoto, Tsuyoshi Araki, Tamao Saito

**Affiliations:** † Graduate School of Science and Technology, 13070 Sophia University, Chiyoda-ku, Tokyo 102-8554, Japan; ‡ Environmental Science Research Institute, Panefri Industrial Co., Ltd., Naha, Okinawa 903-0815, Japan; § Faculty of Science and Technology, 13070 Sophia University, Chiyoda-ku, Tokyo 102-8554, Japan

**Keywords:** *Dictyostelium discoideum*, Meloidogyne incognita, repellent compounds, integrated pest management, sustainable agriculture

## Abstract

Root-knot nematodes are major pests of various crops, coexisting in the soil with amoebae, such as the cellular slime mold Dictyostelium discoideum. Prior studies have indicated that nematodes prey on *Dictyostelium,* prompting investigations into whether amoebae possess chemical defenses against nematodes for potential use in crop protection. Using improved culture and extraction methods, we found that *Dictyostelium* releases metabolites acting as repellents against root-knot nematodes, including L-type basic amino acids (e.g., l-histidine, l-arginine) and antioxidants (e.g., l-ascorbic acid, l-cysteine). A mixture of 14 different repellents proved to be extremely potent. The crude conditioned medium that facilitated the identification of these repellents inhibited root-knot nematode egg hatching, exhibited nematocidal effects, and promoted plant growth in pot tests. These findings suggest that the identified compounds and conditioned medium could provide a novel method for nematode control applicable to crops that lack existing control measures.

## Graphical abstract

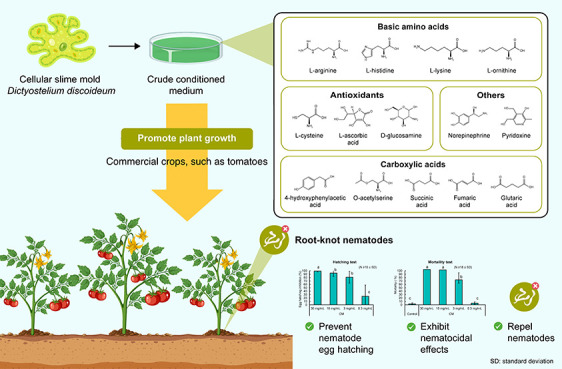

## Introduction

Plant parasitic nematodes are soil-borne pathogens that pose a major threat to global food security. The annual economic losses attributed to these pests are estimated at USD 173 billion.^1^ Root-knot nematodes represent the most important group of plant-parasitic nematodes because of their ability to infect numerous plant species.^2,3^ Among the various species of root-knot nematodes, the southern root-knot nematode (Meloidogyne incognita) is known to infect over 3,000 plant species, many of which are economically important crops.^4^ The most effective method for controlling plant-parasitic nematodes is the application of chemical nematicides.^5^ However, many chemical pesticides exhibit high toxicity, and their application harms the environment and crop producers.^6^ These highly toxic chemical pesticides eliminate nematodes and plant-beneficial soil microorganisms, disrupting the ecosystem and severely impacting soil health.^7,8^ Furthermore, excessive application of these pesticides leads to pesticide resistance.^9,10^ Therefore, establishing novel and effective nematode control methods is an urgent priority for sustainable food production.

Cellular slime molds are soil microorganisms that inhabit forest soils and are characterized by active chemical communication.^11^ Chemical communication among microorganisms primarily depends on low-molecular-weight secondary metabolites, such as polyketides and terpenes secreted by these microorganisms. Genome analysis of the cellular slime mold Dictyostelium discoideum has revealed the presence of more than 40 polyketide synthase and 11 putative terpene synthase genes in its genome.^12,13,14^ Furthermore, concerning polyketide synthase, the enzyme Steely, a fusion of type I and III, is conserved across various species of the cellular slime mold. Conversely, other enzymes are species-specific and have evolved independently;^15,16,17^ this indicates that cellular slime molds possess great potential for producing small-molecule chemicals involved in ecological interactions. The currently known low-molecular-weight compounds of cellular slime molds primarily function as intercellular communication substances that play a role in their morphogenesis.^18,19^

The life cycle of *Dictyostelium* is unique and consists of two distinct stages: unicellular and multicellular. During the unicellular stage, *Dictyostelium* amoebae feed on bacteria and multiply via binary fission. Cells aggregate through chemotaxis to form a multicellular body in response to starvation stimuli that deplete food-bacterial sources. The aggregated cells then form a structure called a mound, within which they differentiate into two cell types: prestalk and prespore cells. The mound eventually becomes a mobile slug that moves via thermotaxis and phototaxis. After the slug stage, the cells differentiate completely to form the final morphology of *Dictyostelium*, the fruiting body. The fruiting body consists of dormant spore cells and vacuolated stalk cells.^20^

Spores serve to transmit life to the next generation. Therefore, spore dispersal is an important survival strategy for cellular slime molds. The spore mass of the fruiting body exists as a sticky mass when moist and a solid mass when dry rather than as fine powder of wind-dispersed spores, thereby hindering its dispersal by wind. *Dictyostelium* is presumed to employ a form of “mediation” for transporting and distributing its spores.^21,22^ From this perspective, *Dictyostelium* has emerged as an excellent model for understanding interspecies communication and cooperation.^23,24^

Free-living nematodes and slime molds occupy the same habitats and share dietary preferences, indicating a close ecological relationship. A 1996 study noted that free-living nematodes prey on amoebas. Furthermore, their dauer larvae were observed climbing the fruiting body of cellular slime molds, possibly attracted to the spores. The adult nematodes congregated around the basal disc of the fruiting body.^25^ These observations strongly suggest that interspecies communication exists between *Dictyostelium* and free-living nematodes.

Therefore, we studied interspecies communication between nematodes and cellular slime molds and discovered that it also exists between root-knot nematodes and cellular slime molds. Previous studies have shown that *Dictyostelium* repels M. incognita and M. hapla and protects plant roots from infection.^26^ In an *in vitro* assay, cell extracts derived from *Dictyostelium* fruiting bodies showed adequate repellent activity to protect plant roots from infection by root-knot nematodes. We found that compounds derived from cellular slime molds could effectively manage the root-knot nematode, a challenging pest to control.

This study aims to enhance sustainable agricultural production by addressing the challenge posed by root-knot nematodes, a recalcitrant pest, through the utilization of methodologies that exhibit reduced environmental impact. To this end, the present study seeks to elucidate the molecular and chemical underpinnings of nematode repellency induced by cellular slime molds, with a focus on identifying the repellent molecules. However, the cell extract used for this purpose had some problems that required improvement. Therefore, we created a conditioned medium (CM) with strong repellent activity in this study and identified its repellent components.

## Materials and Methods

### Nematode Culture

Second-stage juveniles of the root-knot nematode M. incognita were prepared according to a previously described protocol.^27^ Briefly, M. incognita eggs were cultivated with tomatoes (Solanum lycopersicum Pritz). Mature egg masses were collected from the infected tomato roots and incubated in distilled water. The emerged juveniles were collected, washed with a sucrose density gradient followed by water, and stored at 12 °C until further use.

### Cellular Slime Mold Culture and Preparation of Conditioned Medium­(CM)

HMX44A, *amiA*-, and *omt2*- strains were used as developmental defect mutants in this study.^28,29,30^ HMX44A and *amiA*- were obtained from NBRP-nenkin (http://nenkin.nbrp.jp). Given that *omt2*- and *amiA-* strains were developed with an Ax2 background, the Ax2 strain was used as the wild-type strain for assessing repellent activity at each developmental stage. Cells of the wild-type and developmental mutant strains were incubated in the HL-5 medium (Formedium Ltd., UK) until the late growth stage at a density of 6–7 × 10^6^ cells/mL. D. discoideum KAx3 was used as the wild-type strain in all other experiments.

*Dictyostelium* cell extract in ethanol was prepared using KAx3 according to the previously described methodology.^26^ For the CM, KAx3 cells were cultured in a liquid medium, HL-5, until reaching the late growth or stationary phase. In the liquid culture, the cells were cultured by aerating the medium in a tank using a tube rather than the more commonly used shaking culture method. Details are shown in Figure S1. Cells in the late-log or stationary phases in the HL-5 medium were collected, washed, and resuspended in sterilized KK_2_ buffer or sterilized water at a cell density of 1 × 10^8^ cells/mL. After 3 days of static culture, the cells and supernatant were collected as CM. The cell extracts and CM were dried, weighed, and redissolved in 40% methanol to a 100 mg/mL concentration for *in vitro* bioassay analysis, except for the egg-hatching and mortality tests.

### *In Vitro* Bioassays

The nematode chemotaxis assay was performed as previously described.^26^ More than 70% of traces in either area indicated avoidance (Region 2) or attraction (Region 1), depending on which region was favored.^26^ Details are provided in Figure S2.

The plant infection assay was performed according to a previously described protocol.^26^ Details are shown in Figure S3.

To evaluate the repellent activity by cellular slime molds based on developmental stages, cells of the wild-type and developmental mutant strains were incubated in the HL-5 medium, washed with KK_2_ buffer, and resuspended in the same buffer at a concentration of 1 × 10^8^ cells/mL. A 6 cm diameter Petri dish was filled with medium containing 1.5% gellan gum dissolved in KK_2_ phosphate buffer and used as a test plate. Cells were spread at a density of 1.2 × 10^7^ cells/cm^2^ within a 1 × 2 cm rectangle on the test plate. This resulted in a total cell count of 2.4 × 10^7^ cells. After 24 h, when each strain reached its final morphology, 10–15 nematodes were placed at the center of a Petri dish 1.2 cm away from the cellular slime mold. After 16 h, snapshots of their movement were obtained using a microscope (AZ100, Nikon, Japan) equipped with a camera (FR-400C, Flovel, Germany) and analyzed as described Figure S2.

In the egg hatching test and mortality test, nematodes were cultured in aqueous solution. Hence, CM dissolved in 40% methanol was not used, and CM was adjusted to each concentration using the original solution and water. The concentration of the original CM solution was determined by drying and weighing the sample. Water was used as the control. In these two types of tests, CM was used at concentrations of 30 mg/mL, 15 mg/mL, 3 mg/mL, and 0.3 mg/mL. The concentration of 30 mg/mL signified undiluted CM. Egg-hatching and juvenile mortality tests were performed according to previous reports with minor modifications.^31,32^ One mL of CM adjusted to each concentration was introduced into a 24-well plate, and 60 eggs were added in each well. Then the exact number of eggs was counted. Plates were incubated at room temperature (22 °C) for 3 days, and the number of hatched juveniles was counted. Five replicates were used for each concentration in each experiment, and each experiment was repeated three times.

In the mortality test, 30 nematode juveniles were mixed with CM, diluted with water to each concentration, and placed in wells of a 96-well plate. The total volume of each sample was set to 100 μL. Six replicates were used for each concentration in each experiment, and the experiments were repeated three times. Postmixing, the number of nematodes was counted. After a 2-day incubation period, a drop of 1 M NaOH was added, and specimens that twisted or curled within 2 min were counted as alive. The eggs and hatched juveniles were counted under a microscope (SMZ 745T, Nikon, Japan). Egg-hatching and mortality rates were calculated as previously reported.^31,32^

### Plant Root Protection Assay in the Pot: Root-Knot Index and Control Value

Twelve soil-based experiments were conducted between 2021 and 2022; in one experiment, 3–5 pots were used for treatments. Pot tests were conducted using Wagner pots. Each Wagner pot has a capacity of 4 L, and 3,000–5,300 nematodes were inoculated into 4 L of soil. The soil used was from the test field of the Environmental Science Research Institute, Panefri Industrial Co., Ltd. One tomato seedling was planted in each pot, and in addition to regular watering, 150–200 mL of diluted CM was applied each time. In the control group, water was used instead of CM. The experimental period ranged from 34 to 63 days. In other words, tests were conducted for one month and two months. Subsequently, the plant roots were excavated and divided into sections near the aboveground parts, followed by weight measurement. The underground parts were evaluated for nematode infection using the 0–4 scale of the root-knot index.^33^ The root-knot index was evaluated based on the following characteristics, and the average value of 3–5 Wagner pots was calculated each time:

0: No root knot.

1: Root knot is slightly visible but not noticeable.

2: Root knot is briefly visible. A few large or connected root nodules exist.

3: Numerous large and small root nodules are visible. Root nodules thicken a portion of roots, constituting less than 50% of the total root area.

4: Numerous roots are thickened by root nodules.

The control value was calculated using the following formula:

Controlvalue=100−(averageroot‐knotindexofsample/averageroot‐knotindexofcontrol)×100

### Metabolomic Analysis

The CM underwent two-phase separation via the Bligh–Dyer method.^34^ The metabolomic analysis of the water-soluble components of the CM was performed by the Chemicals Evaluation and Research Institute in Japan. Detailed method is described in Table S2.

### Statistical Analysis

The data were expressed as mean ± standard deviation (SD) and analyzed using R version 4.1.2. The minimum number of replicate samples was 9, and the numbers of replicate samples are depicted in Figures 2–8. All statistical analyses were conducted using the nonparametric multiple comparison Steel–Dwass test. The level of significance was set at *p* < 0.05.

## Results and Discussion

### *Dictyostelium* Conditioned Medium (CM) Repels Plant Parasitic Nematodes

Cell extracts from D. discoideum were observed to repel the root-knot nematodes M. incognita and M. hapla.^26^ However, the preparation of cell extracts had several limitations. First, cell culture was time-consuming and labor-intensive, particularly the formation of fruiting bodies on agar plate media. Second, the use of organic solvents during the extraction process was undesirable. The critical point was the exceedingly low yield of 1 mg from 1 × 10^8^ cells. To address these problems, we reexamined repellent activity across developmental stages. The previous method used a wild-type strain that required 48 h incubation on an agar plate for complete fruiting body formation, whereas the amoeboid stage necessitated only 2–3 h of incubation.^26^ Consequently, we considered the potential inadequacy of repellent component release. Three mutants exhibiting developmental defects were used for this examination. The HMX44A and *omt2-* mutants exhibited a mound-arrest phenotype, while the *amiA-* mutant was characterized by an absence of aggregation (Figure 1).^28,29,30^

**Figure 1:**
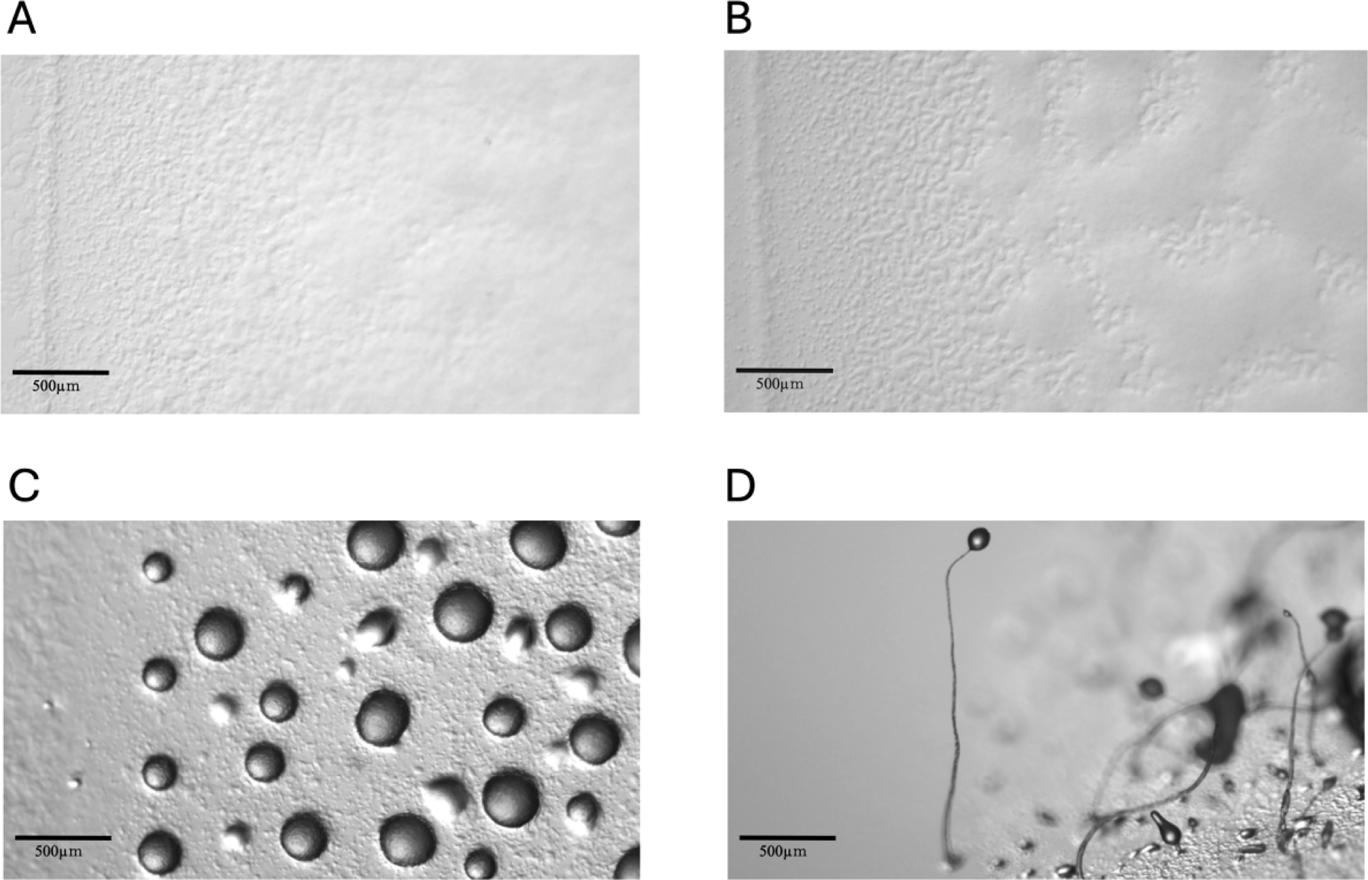
Developmental morphology of mutant strains and wild type strain. Cells were incubated in the HL-5 medium until the late growth stage at a density of 6–7 × 10^6^ cells/mL, washed with KK_2_ buffer and inoculated on KK_2_ buffered agar plate. Each photo was taken 24 h poststarvation (t24) with microscope (SMTZ745T, Nikon, Japan) and digital camera (E31SPM, TopTek, China). (A) The *amiA-* strain showed aggregation-less phenotype. (B) The HMX44A strain ceased development at the loose-mound stage. (C) The *omt2-* strain showed tight-mound arrest. (D) The wild-type Ax2 strain forms a fruiting body at t24.

The results of the *in vitro* bioassay to assess nematode behavior are summarized in Figure 2A,B. After 24 h of morphogenesis, all three mutants showed repellent activity against M. incognita comparable to that of the fruiting bodies of the wild-type strain. Based on these results, fruiting body formation on agar plates was deemed unnecessary. The culture method was altered from a two-membered culture method using SM agar plates to a liquid culture method using the synthetic medium HL-5, and CM was obtained. The nematode repellency activity of cell extracts and CM obtained from this method was compared. CM exhibited comparable or superior repellent activity relative to the cell extract (Figure 2C,D). Five milligrams of CM showed repellent activity equivalent to 8 mg of cell extract. Furthermore, cell extracts showed a yield of 1 mg per 1 × 10^8^ cells, whereas CM showed an average yield of approximately 30 mg for the same number of cells, thereby indicating a substantial increase in the yield. Based on these data, we decided to use the CM instead of cell extracts for further experiments.

**Figure 2:**
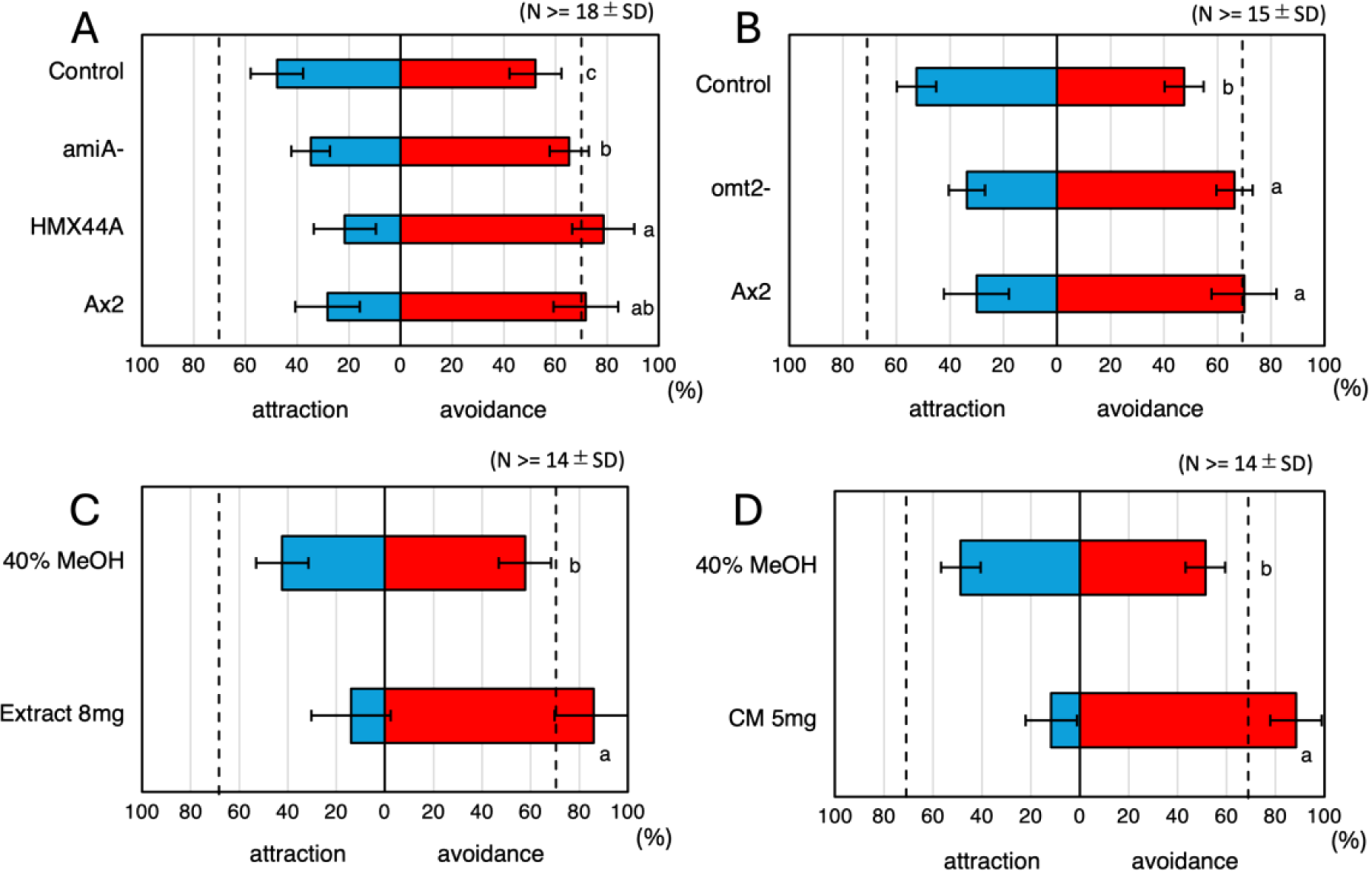
Repellent activity of each strain and repellent activity of the conditioned medium (CM). (A) The trace amounts on the avoidance side (Region 2) of the mutants *amiA-* and HMX44A are significantly different from those in the control (KK_2_ phosphate buffer) and not significantly different from those observed in the Ax2 fruiting body. Values are expressed as mean ± SD. (B) The trace amount on the avoidance side (Region 2) of the *omt2*- mutant is significantly different from that in the control (KK_2_ phosphate buffer) and not significantly different from that observed in the Ax2 fruiting body. (C) The trace in the avoidance side of 8 mg cell extract is significantly different from that in the control (40% methanol). (D) The trace in the avoidance side of 5 mg of CM is significantly different from that in the control (40% methanol). The amount of traces on avoidance side is indicated by a red bar, and the amount of traces on the attractant side is indicated by a blue bar. Different letters denote significant differences between groups at *p* < 0.05.

### CM Inhibits Egg-Hatching and Juvenile Survival

*In vitro* egg-hatching and juvenile mortality tests were performed to investigate the biocontrol capability of the CM against M. incognita. Figure 3A presents the results of the egg-hatching test. The concentration of 30 mg/mL signified undiluted CM. In this case, it indicated 99% hatching inhibition. The 15 mg/mL or 2× dilution of the CM showed 93% inhibition, whereas the 3 mg/mL or 10× dilution exhibited 81% inhibition. Conversely, the 100-fold dilution of 0.3 mg/mL revealed only 25% inhibition. Figure 3B presents the mortality test results. The 30 mg/mL CM and 15 mg/mL CM eradicated 100% of root-knot nematodes; the 3 mg/mL CM eliminated 71%. The 0.3 mg/mL CM showed no significant difference from the control water. These data indicate that CM can eliminate nematodes at high concentrations and inhibit egg-hatching; 30 and 15 mg/mL CM inhibit both simultaneously; 3 mg/mL CM inhibits egg-hatching by 81% and eliminates 71% of root-knot nematodes.

**Figure 3:**
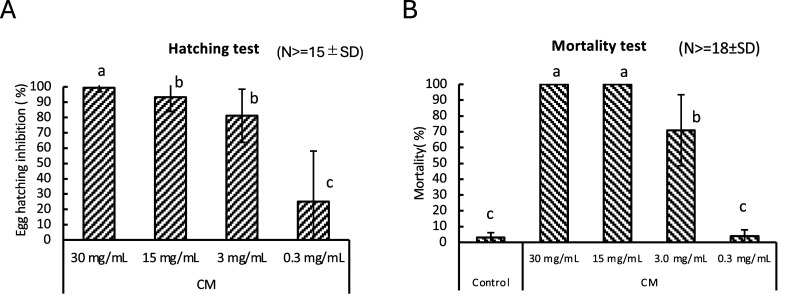
Biocontrol capacity of CM. (A) The result of the hatching inhibition test. High concentrations of the CM showed hatching inhibition in root-knot nematodes. CM 30 mg/mL exhibited 99% hatching inhibition. CM 15 mg/mL demonstrated 93% inhibition, whereas the 3 mg/mL exhibited 81% inhibition. CM 0.3 mg/mL showed 25% hatching inhibition. Values are expressed as mean ± SD of *N* ≥ 15. Different letters denote significant differences between groups at *p* < 0.05. Error bars are constrained within the possible range of 0% to 100%. (B) The result of the mortality test. Concentrations of 30–3 mg/mL CM were highly lethal to root-knot nematodes. Water served as the control. CM 0.3 mg/mL showed no significant difference from control.

### CM Can Protect Plant Roots from Root-Knot Nematode Infection and Facilitate Plant Growth

We used Wagner pots to investigate the ability of CM to protect plant roots from nematodes in the soil. Tests to determine whether effective control can be achieved in soil are essential for field trials, and the use of Wagner pots enables estimation of the amount of CM to be used in the field.Table S1 is a summary of Wagner pot soil experiments for one month. When the control value exceeded 50%, effective control was achieved. As shown in Table S1, daily processing of 1.2 g of CM per plant for approximately 40 days, corresponding to a one-month test, demonstrated a pest control value of 50% or higher, stably controlling nematodes and protecting plants. This condition, which involves daily processing of 1.2 g of CM per plant, was confirmed to be effective not only during a one-month culture period but also over a two-month, as shown in Table 1; this means that CM alone was able to significantly protect plant roots for two months under conditions of high-density distribution, where 5,000 nematodes were present in 4 L of soil. Furthermore, it also enhanced the growth of the plant’s aboveground parts, specifically the parts used as the crop (Tables 1 and S1).

**Table 1:** Summary of Two Months Wagener Pot Experiment^a^

Sample	Number of times processed per week	Weight of dry CM and amount of water used per plant in a single treatment (g/mL)	Above ground weight after application (g)	Average weight (g)	Root-knot index	Average of root-knot index	Post control value	Application period (number of inoculated nematodes)
control-1	7/week	0 g/150 mL	218	229	4	4	-	63 days(5000)
control-2	7/week	0 g/150 mL	224		4		-	
control-2	7/week	0 g/150 mL	246		4		-	
CM1-1	7/week	2.4 g/150 mL	540	505	1	1	75	
CM1-2	7/week	2.4 g/150 mL	471		1			
CM1-3	7/week	2.4 g/150 mL	505		1			
CM2-1	7/week	1.2 g/150 mL	487	478	1	1.3	66.7	
CM2-1	7/week	1.2 g/150 mL	423		1			
CM2-1	7/week	1.2 g/150 mL	524		2			

a The number of times processed per week: 7/week means that the treatment was performed every day. In each treatment, CM was dissolved in 150 mL of water at a dry weight of 2.4 or 1.2 g and applied to the roots of the plants by drip irrigation. In the control group, only 150 mL of water was applied. In this experiment, 5000 nematodes were inoculated into each pot containing 4 L of soil.

### Repellent Compounds in *Dictyostelium* CM

An *in vitro* chemotaxis assay demonstrated that M. incognita J2s significantly repelled *Dictyostelium* CM compared with the 40% methanol control (Figure 2C,D). These results suggest that the compounds in *Dictyostelium* CM repel root-knot nematodes. Isolating and identifying chemical compounds crucial in repelling root-knot nematodes elucidate the mechanisms underlying the interactions between root-knot nematodes and cellular slime molds in the soil and provide insights into the molecular interactions for controlling root-knot nematodes.

As a first step in analyzing the repellents, the CM was subjected to a two-phase separation of the organic solvent and water using the Bligh–Dyer method.^34^ Excluding the fluff layer, an average of 57.9% and 8.3% of the initial weight was recovered in the aqueous and organic phases, respectively. The repellent activity of each fraction was examined using an *in vitro* chemotaxis assay and repellent activity was detected in the aqueous phase but not in the organic phase. The repellent activity was tested using 2.95 mg of the aqueous phase and 0.48 mg of the organic phase derived from 5 mg of the CM. As shown in Figure S4, 2.95 mg of the aqueous phase exhibited an effect equivalent to that of 5 mg of the CM. However, no significant difference was observed between the organic phase and the control (40% methanol). Based on these data, we decided to use the aqueous phase of *Dictyostelium* CM for further analysis and subjected it to metabolomic analysis. Metabolome analysis detected 126 compounds in the aqueous phase of CM. Of the 20 proteinogenic amino acids, 19 were included among the 126 compounds. Only lysine was undetected; however, its repellent activity was also examined. Therefore, the repellent activities of all 127 purchased compounds were examined using an *in vitro* chemotaxis assay (Table S2). Consequently, 14 compounds exhibited repellent activity. They can be classified into four principal groups: basic amino acids, antioxidants, carboxylic acids, and other less-defined categories. The details of each group are summarized in Figure 4.

**Figure 4:**
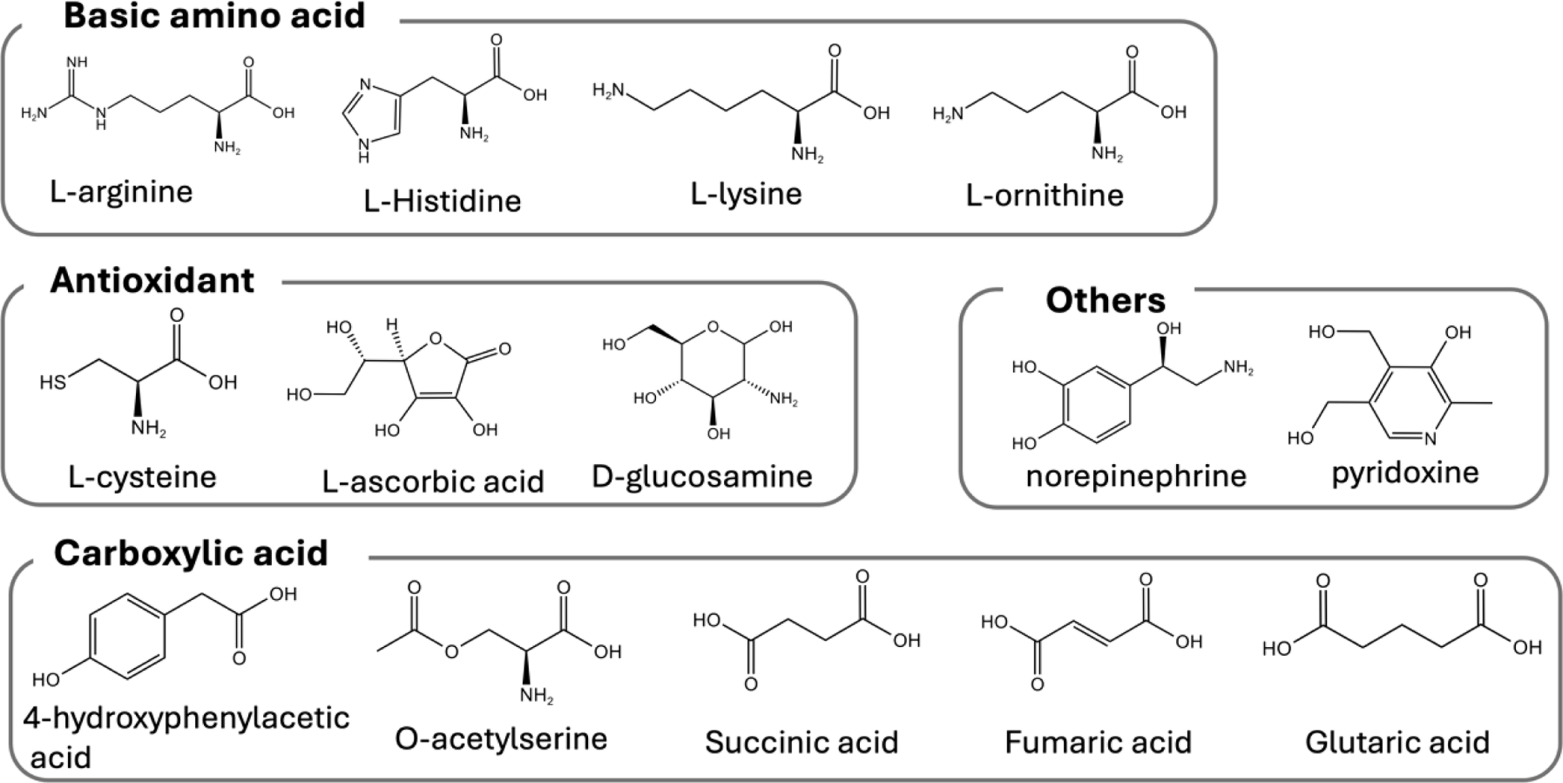
Chemical structures of identified 14 repellent compounds. The 14 chemicals identified were classified into four groups based on their biological functions: basic amino acids, antioxidants, carboxylic acids, and others.

The repellent activities of the four basic amino acids are shown in Figure 5A–D. *Dictyostelium* CM (5 mg) was used as a positive control, whereas methanol (40%) was used as a negative control. The four basic amino acids exhibited concentration-dependent repellent activity, with each demonstrating repellent activity equivalent to the positive control (5 mg of CM at a quantity of 0.1 mg). In other words, no statistically significant difference was observed in the repellent activity between 5 mg of the positive control and 0.1 mg of the amino acid; this indicates that these four amino acids possess repellent activities that are 50 times stronger than the CM.

**Figure 5:**
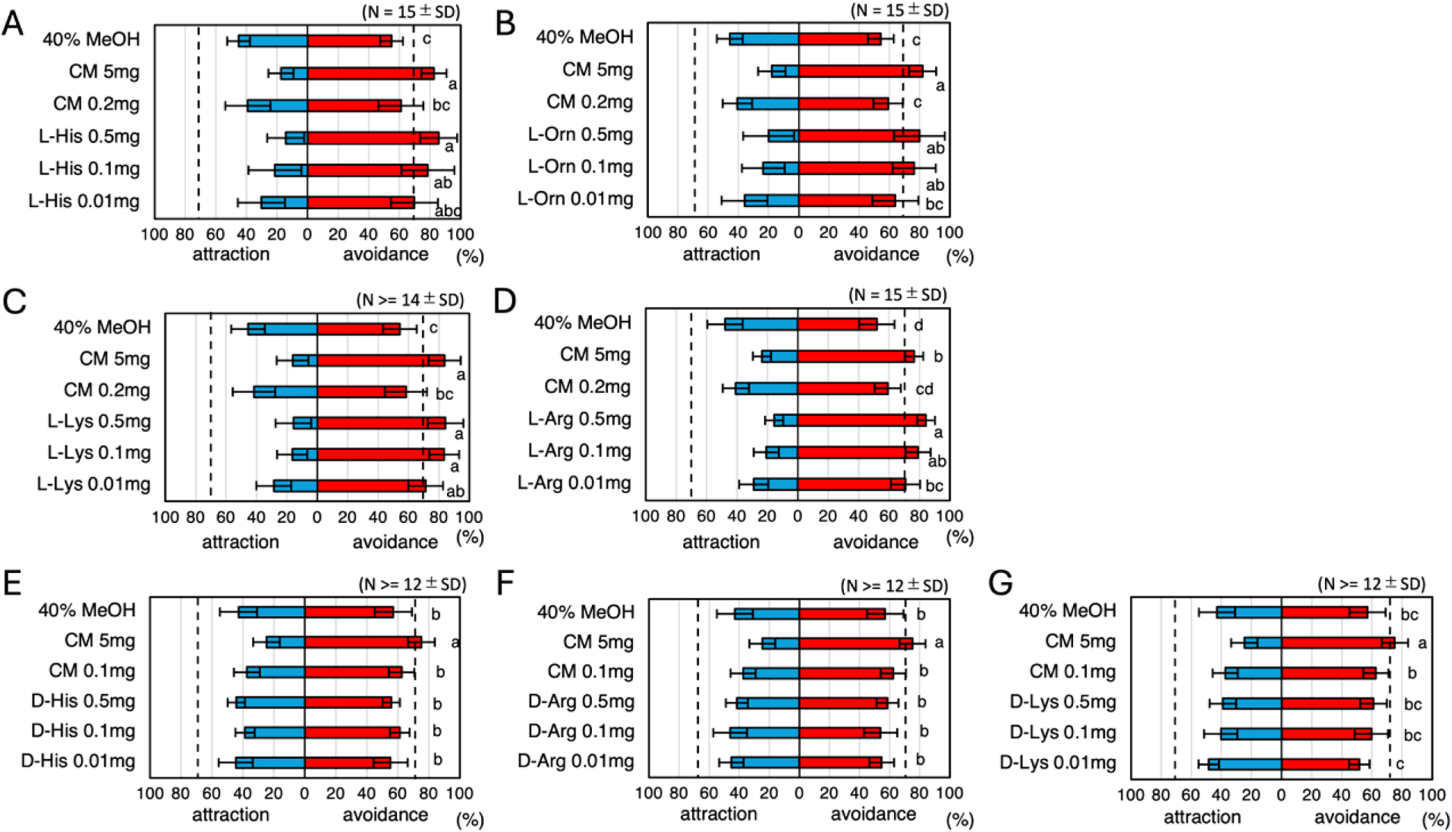
Repellent activity of identified compounds: amino acids. (A–D) Repellent activity of four L-type basic amino acids. The dose-dependent repellent activity could be observed; all four amino acids were not significantly different or more repellent than 5 mg CM up to 0.1 mg. l-His, l-histidine; l-Orn, l-ornithine; l-Lys, l-lysine; l-Arg, l-arginine. (E–G) Repellent activity of D-type basic amino acids. D-type basic amino acids showed no repellent activity. The repellent activity of each amino acid was significantly different from 5 mg CM and showed no significant difference from 40% methanol. d-His, d-histidine; d-Arg, d-arginine; d-Lys, d-lysine. The amount of traces on avoidance side is indicated by a red bar, and the amount of traces on the attractant side is indicated by a blue bar.

Given that these four amino acids were L-type, we also examined the repellent activity of D-type basic amino acids to verify their specificity. Figure 5E–G illustrates that D-type basic amino acids showed no repellent activity; this indicates that only L-type basic amino acids can repel root-knot nematodes, whereas D-type basic amino acids cannot.

Previous reports have shown that NH_4_^+^ exhibits a strong repellent effect against M. incognita.^35^ Therefore, the repellent effect of basic amino acids might be attributed to the amino group in the side chain. However, since no repellent effect was observed in D-type amino acids, it became clear that this was not simply due to the amino group. Conversely, dl-methionine has been shown to inhibit the hatching and mobility of M. incognita J2 and eggs in laboratory experiments;^36^ this is attributed to the inhibition of metabolic processes or enzymes by dl-methionine *in vivo*. However, no repellent effect was observed with D-basic amino acids in our study. Among the basic amino acids, l-arginine has been reported to cause a 100% mortality rate after 7 days of exposure at a concentration of 2,000 ppm (2 mg/mL).^37^ In our assay, the four L-type basic amino acids have equivalent repellent activity.

Figure 6A–C shows the repellent activity of three antioxidant compounds.^38,39^ l-Cysteine and d-glucosamine exhibited the strongest repellent activities at 0.1 mg. In contrast, l-ascorbic acid demonstrated repellent activity over a wide range, with repellent activity at 0.005 mg. l-Cysteine and d-glucosamine exhibited approximately 50-fold activity compared to the control, whereas l-ascorbic acid showed up to 1,000-fold activity compared to the control. The repellent activity of the ascorbic acid isomer was also verified. Figure 6D illustrates the results of the chemotaxis assay for the ascorbic acid isomer. Only d-iso-ascorbic acid was obtained; however, it did not exhibit repellent activity, whereas l-ascorbic acid demonstrated repellent activity. Ascorbic acid, like l-arginine, has been reported to have a lethal effect on M. javanica.^37^ The results indicate basic amino acids and ascorbic acid were active in the L-form but not in the D-form; this may suggest that the stereostructure of these compounds plays a role in their reception or signal transduction of repellency.

**Figure 6:**
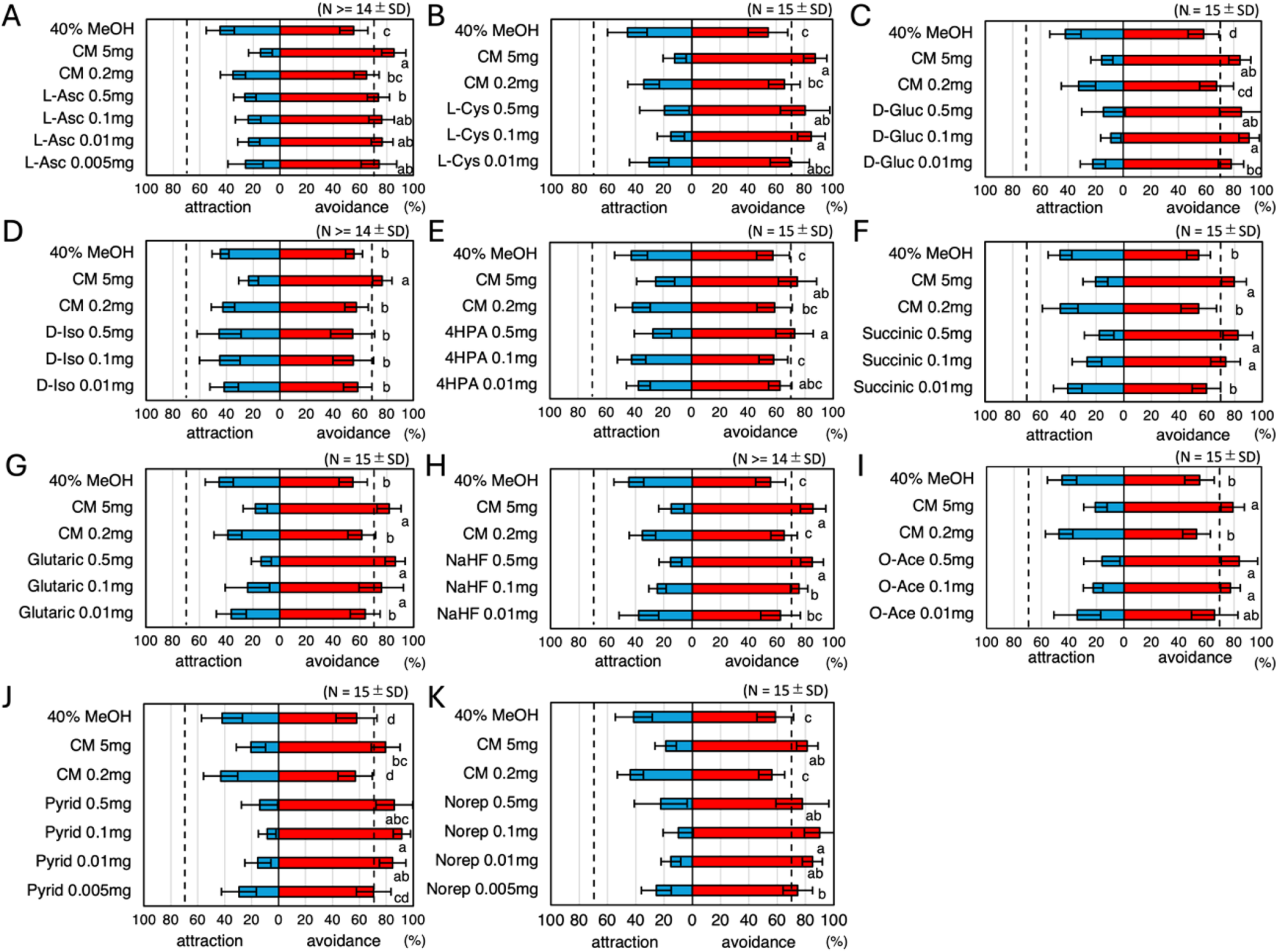
Repellent activity of identified compounds: antioxidants, carboxylic acids, and others. (A–C) Repellent activity of antioxidant compounds. l-Ascorbic acid was significantly different from the negative control (40% methanol) from 0.5 mg up to 0.005 mg. l-Cysteine was significantly different from the negative control up to 0.1 mg. d-Glucosamine was significantly different from the control up to 0.01 mg. L-Asc, l-ascorbic acid; l-Cys, l-cysteine; d-Gluc, d-glucosamine. Values are expressed as mean ± SD. Different letters denote significant differences between groups at *p* < 0.05. (D) The isomer of l-ascorbic acid, D-isoascorbic acid was not significantly different from the control (40% methanol). d-Iso, d-isoascorbic acid. (E–I) Five compounds considered carboxylic acids also exhibited concentration-dependent repellent activity. Only 4-hydroxyphenylacetic acid significantly differed from the negative control at 0.5 mg, not 0.1 mg. The other four compounds, succinic acid, glutaric acid, fumaric acid, and O-acetylserine, were significantly different from the control up to 0.1 mg and judged to possess repellent activity. (J,K) Pyridoxine and norepinephrine exhibited strong repellent activities and were significantly different from the control within the range of 0.5–0.005 mg. 4HPA, 4-hydroxyphenylacetic acid; Succinic, succinic acid; Glutaric, glutaric acid; NaHF, fumaric acid (sodium hydrogen fumarate); O-Ace, O-acetylserine; Pyrid, pyridoxine; Norep, norepinephrine. The amount of traces on avoidance side is indicated by a red bar, and the amount of traces on the attractant side is indicated by a blue bar.

Five compounds were identified as carboxylic acids: 4-hydroxyphenylacetic acid, O-acetylserine, succinic acid, fumaric acid, and glutaric acid. Figure 6E–I shows the repellent activity of each compound. All the compounds showed concentration-dependent repellent activity. Moreover, 0.5 mg of 4-hydroxyphenylacetic acid and fumaric acid exhibited equivalent repellent activity to 5 mg of CM. Furthermore, 0.1 mg of the other three compounds exhibited repellent activity equivalent to the positive control. 4-Hydroxyphenylacetic acid and fumaric acid exhibited 10-fold stronger activity, while the other three compounds showed 50-fold stronger repellent activity than the control.

Carboxylic acids are known to have antimicrobial activity.^40^ One aspect of this antimicrobial activity is their ability to maintain a low pH. Still, because the root-knot nematode M. incognita prefers a low pH of 4.5–5.4,^41^ the repellent activity of carboxylic acids against nematodes is unlikely due to pH. Among carboxylic acids, 4-hydroxyphenylacetic acid has been reported to have nematocidal activity.^42^

Furthermore, dicarboxylic acids such as fumaric acid and succinic acid have been reported to exhibit toxicity against nematodes regardless of pH. It appears that the spatial configuration of these compounds is involved in their toxicity.^43^

Finally, we examined the repellent activity of the “Other” group. This group included pyridoxine and norepinephrine. As shown in Figure 6J,K, 0.01 mg of pyridoxine and norepinephrine exhibited equivalent repellent activity with 5 mg of control CM; this indicates that these two compounds exhibit repellent activity 500-fold stronger than that of the control. Caenorhabditis elegans has been reported to lack norepinephrine signaling; therefore norepinephrine is likely to be an unknown substance to nematodes.^44^

These results suggest that *Dictyostelium* CM exhibits repellent activity owing to multiple relatively mild active repellent components. Most known natural repellent compounds are volatile organic compounds that induce avoidance through weak toxicity.^45^ In such cases, nematodes use olfactory neurons to detect volatile compounds.^46,47^ However, the compounds found in this study are mostly primary metabolites essential to living organisms. Root-knot nematodes use chemical cues (tastes) to detect their host plants.^48,49^ Similarly, they may use chemical signals to avoid harmful stimuli. The identified repellent substance may mimic cues associated with natural enemies.

### *In Vitro* Inhibition of Plant Infection by Nematodes

We next investigated whether these compounds exhibited repellent activity even in the presence of plant roots that secrete exudates, thereby attracting nematodes and protecting the roots from nematode infection.^50^ In the plant infection assay, 5 mg of the CM served as a positive control, while the repellent compound was used at one-tenth of that amount, 0.5 mg.

To verify how far the infection-inhibitory effects of the compounds extend, we examined the effects on plants in positions I–III (Figure 7A–C). However, plants in positions II and III did not show significant difference versus control. Therefore, we compared the infection-inhibitory effects on the plants in position I.

**Figure 7:**
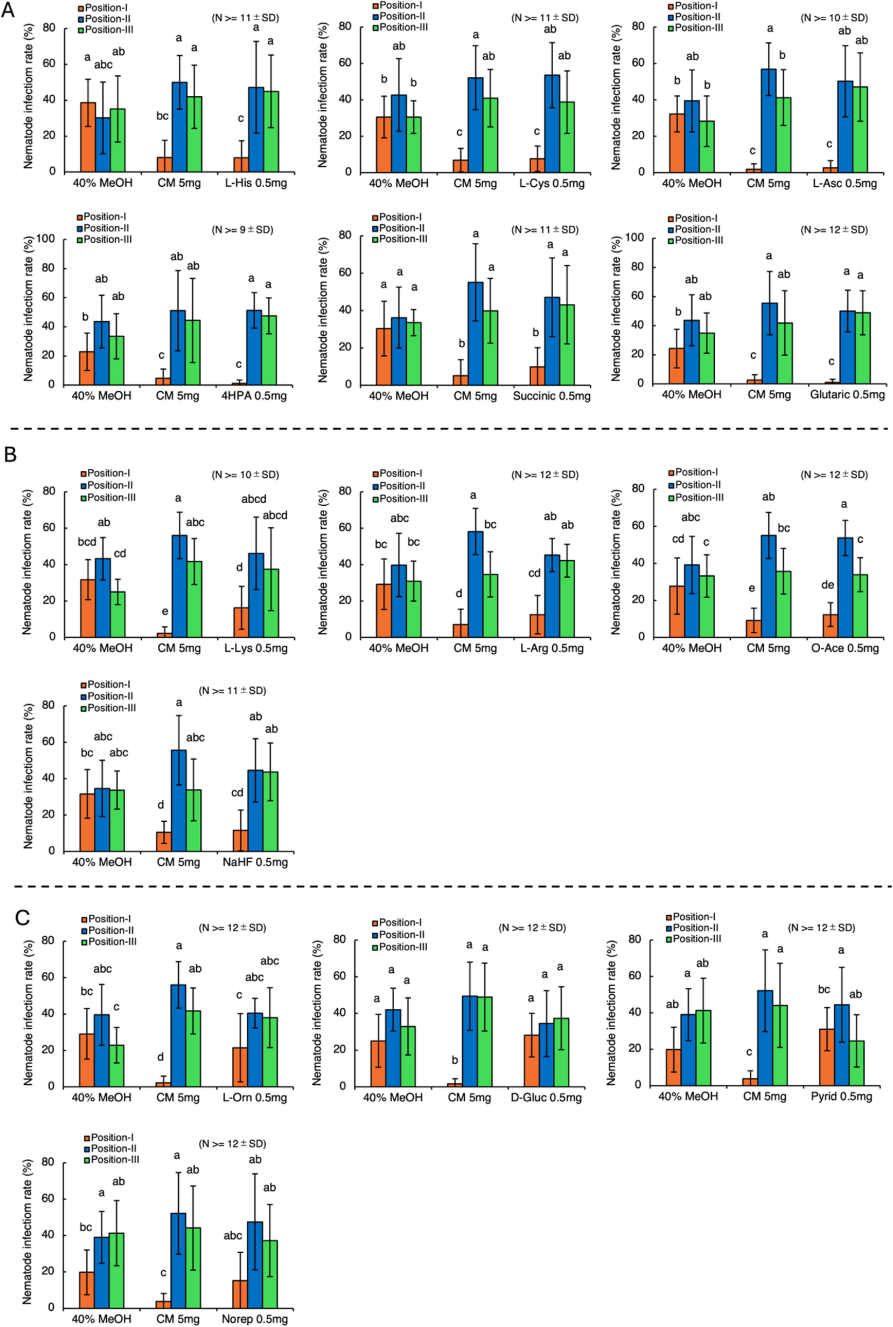
Plant protection by repellent compounds. (A) Comparing the percentage of infected plants in Position I, six compounds, namely l-histidine, l-cysteine, l-ascorbic acid, 4-hydroxyphenylacetic acid (4HPA), succinic acid, and glutaric acid, displayed plant root protection activities at 0.5 mg, equivalent to 5 mg CM, and were significantly different from the control (40% methanol). (B) Comparing the percentage of infected plants in Position I, 0.5 mg of l-lysine, l-arginine, O-acetylserine, and fumaric acid (sodium hydrogen fumarate) were intermediate in activity between the control, 40% methanol, and 5 mg CM, with no significant differences. (C) Comparing the percentage of infected plants in Position I, 0.5 mg l-ornithine, d-glucosamine, pyridoxine (pyridoxine hydrochloride) and norepinephrine showed no significant difference to the control or rather weak plant protection activity.

Fourteen identified repellent compounds were evaluated for their ability to inhibit nematode infection in plants. Consequently, six compounds demonstrated plant infection inhibition equivalent to or greater than that of CM 5 mg: l-histidine, l-cysteine, l-ascorbic acid, 4-hydroxyphenylacetic acid, succinic acid, and glutaric acid (Figure 7A). l-lysine, l-arginine, O-acetylserine, and fumaric acid (sodium hydrogen fumarate) were considered to provide protection to plant roots, albeit less effectively than the 5 mg CM control (Figure 7B). l-ornithine and norepinephrine showed weak inhibitory effects against infection. Furthermore, because d-glucosamine and pyridoxine exhibited no significant difference from the negative control at position I, they were regarded as lacking a plant infection-inhibitory effect (Figure 7C).

Some compounds showed repellent activity against root-knot nematodes, but their activity in suppressing plant infection was low. The effect of inhibiting plant infection is more complex than that of repellent activity; therefore, compounds with strong infection inhibition activity should be prioritized for further experiments due to their relevance to actual field conditions.

### Synergistic Effect of the Repellent Compounds

Given that the *Dictyostelium* CM comprises at least 14 repellent compounds, we subsequently investigated the potential synergistic effect of these repellent components on repellent activity. We assessed the potential synergistic effect of combining the four basic amino acids. Figure 8A illustrates the absence of a synergistic effect in the mixture of the four amino acids. The four basic amino acids each showed repellent activity equivalent to 5 mg of CM at 0.1 mg each. A mixture of the four basic amino acids showed activity equivalent to 5 mg CM at 0.1 mg. This result is the same as when the repellent activity of amino acids was examined individually. In other words, no synergistic effect was observed. However, an increase of repellent activity was observed when one compound from another category was mixed with the four amino acids (Figure 8B,C). In this case, 0.5 mg, 0.1 mg, and 0.01 mg of a mixture of the four amino acids and one compound from another category in equal proportions were used. In particular, l-ascorbic acid exhibits strong repellent-enhancing activity. Subsequently, we created mixtures of 13 and 14. Mix14 was a mixture of all 14 repellent components. l-lysine was not detected in metabolome analysis; therefore, Mix 13 was a mixture of all components except lysine. In both cases, the compound mixture exhibited more than 100 times the repellent activity level compared to the CM (Figure 8D,E); 0.05 mg of Mix14 or Mix13 was equivalent to 5 mg of CM in terms of activity, and 0.01 mg of Mix14 or Mix13 showed no significant difference from 5 mg of CM.

**Figure 8:**
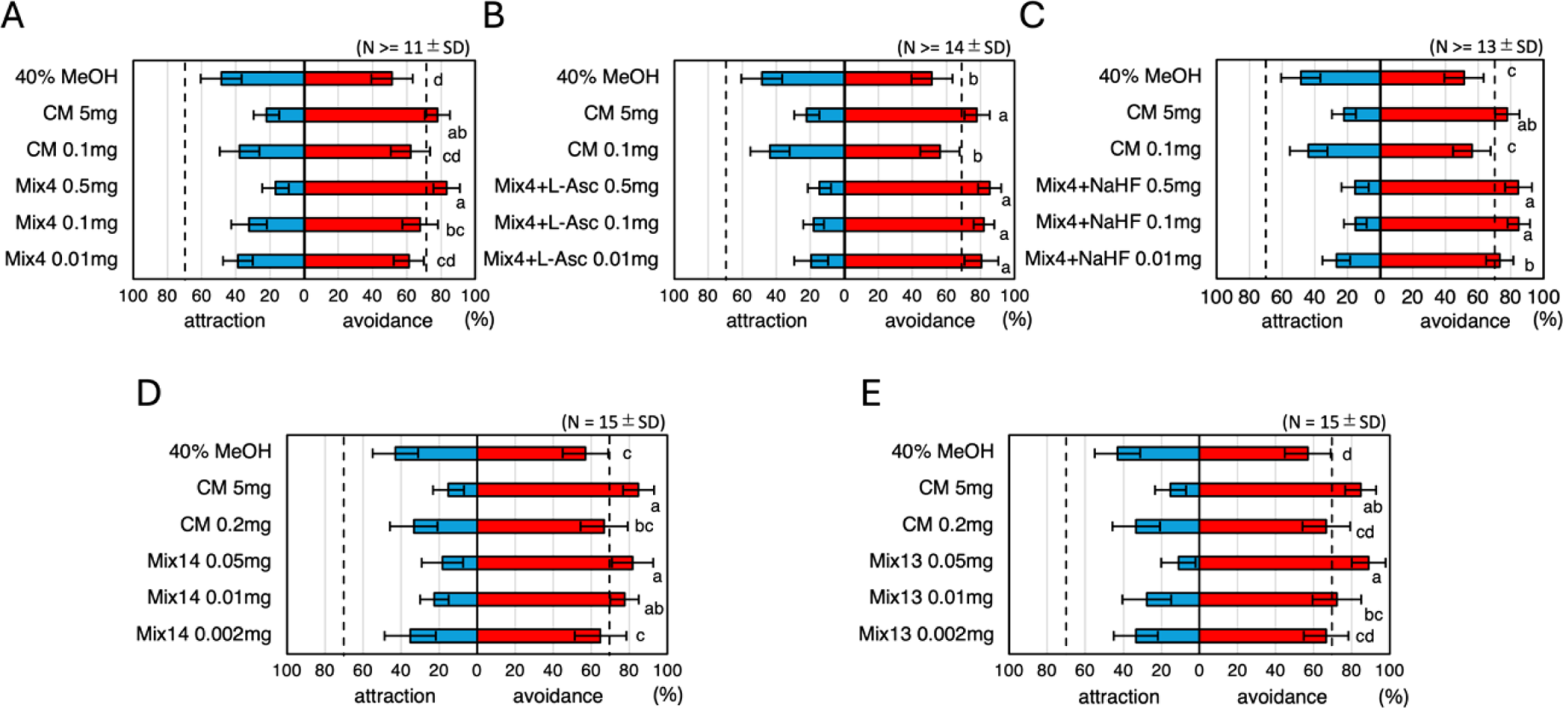
Equal-weight mixture of repellent compounds demonstrates strong repellent activity. (A) A mixture of four basic amino acids (labeled Mix4) in equal amounts of 0.5 mg showed no significant difference from CM 5 mg. (B) When ascorbic acid was added to the four basic amino acids (labeled Mix4+L-Asc), even at a total concentration of 0.01 mg, it exhibited repellent activity comparable to 5 mg of CM. (C) The same applied when fumaric acid was added (labeled Mix4+NaHF). (D,E) The repellent effect of mixing equal amounts of 13, and 14 (indicated as Mix13, and Mix14) of the 13 and 14 repellent compounds. A total of 0.01 mg showed equivalent repellent activity to 5 mg of CM in both cases; this indicated that, when mixed, 1/500th of the amount exhibited activity equivalent to that of the CM. l-Asc, l-ascorbic acid; NaHF, fumaric acid (sodium hydrogen fumarate). The amount of traces on avoidance side is indicated by a red bar, and the amount of traces on the attractant side is indicated by a blue bar.

Mixtures of multiple repellent compounds may exhibit synergistic or complementary effects on repellent activity. Reports have indicated that fumigant and nonfumigant pesticide combinations synergize in controlling root-knot nematodes.^51^ According to the authors, a single pesticide is no longer reliable due to pest resistance. Another well-known example of the synergistic effect of natural compounds is an essential oil that repels mosquitoes, thereby reducing the risk of mosquito-borne diseases, such as dengue.^52^ The repellent efficacy of essential oils can be improved by combining several essential oils.^53^

In this study, we established a method to obtain root-knot nematode repellent activity derived from cellular slime mold in the form of CM, which enables large-scale cultivation and eliminates the need for extraction using organic solvents. This approach reduces environmental impact and allows for the identification of repellent compounds without concerns about byproduct generation from organic solvents. Some of these identified repellent compounds have already been reported to have nematocidal effects.^37,42,43^ Still, the identification of repellent compounds has opened the possibility of reducing the amount of CM used in future field trials. Furthermore, understanding the repellent compounds has enabled us to examine the repellent mechanism of root-knot nematode. Since synergistic repellent effects were observed when multiple repellent compounds were mixed, these compounds may enhance repellent behavior by utilizing multiple different signaling pathways. Research is progressing on the chemical perception and signal transduction of compounds that control these behaviors in C. elegans.^44^ Based on these research results, it is possible to analyze root-knot nematodes. It is important to verify at the genetic level how repellent substances induce repellent behavior in root-knot nematodes, and this is the next step of our study.

By identifying genes involved in repellent signal transduction pathways, we can develop high-throughput analytical methods using these genes, enabling the screening of new repellent substances and the design of safer substances with higher repellent activity. In this study, we utilized the water-soluble fraction of CM; however, the possibility of repellent compounds remaining in the organic fraction cannot be ruled out. This issue is expected to be resolved by employing high-throughput assay methods to identify repellent substances. We examined the repellent effect and plant protection activity of CM based on the behavior of live nematodes. However, verifying the behavior of living organisms poses many challenges, including large fluctuations in numerical values and the difficulty of processing large numbers of samples. However, identifying genes involved in signal transduction of repellent activity will enable verification at the genetic level and contribute to solving these challenges.

Cellular slime molds inhabit the soil, which is a complex environment. However, ecological research on them is still in its infancy. It has been discovered that they engage in complex chemical communication with bacteria that serve as their food rather than simply a predator–prey relationship.^54^ Transporting spores is a crucial aspect of their survival strategy. The possibility of spore transport by fruit flies, birds and insects has been suggested.^21,22^ A 1996 report suggested that free-living nematodes transport spores by consuming them. Since cellular slime molds live in the top 1.5 cm of soil,^20^ we hypothesize that they actively avoid plant-parasitic nematodes that burrow deep into the soil, as this would be disadvantageous.

Plant-parasitic nematodes are obligate parasites; their survival depends on their ability to parasitize host plants. The *Dictyostelium* CM reduces crop damage by repelling nematodes, with mild effects. It cannot be expected to have the same strong effect as the fumigants currently in use, which eradicate root-knot nematodes before planting. However, CM and repellent compounds can be used during the crop growth period, and their continuous use can gradually reduce the number of root-knot nematodes in the soil. Some issues need to be addressed in the future regarding mass production and costs; however, CM and repellent compounds derived from cellular slime molds can contribute to sustainable food production and improved soil health as part of an integrated pest management approach.

## Supplementary Material



## Notes

The diagram of large-scale culture and CM preparation method, schematic diagrams of *in vitro* chemotaxis assay and *in vitro* plant infection assay, repellent activity of water-soluble repellent compounds, results of one month Wagner pot experiments, metabolomic analysis of CM (list of 127 compounds) (PDF)
