# Supplementary material for: Identification of Slime Mold Metabolites That Confer Protection to Commercial Crops against Root-Knot Nematodes
Source: J Agric Food Chem. 2025 Jul 29;73(31):19315–26. doi: 10.1021/acs.jafc.5c04345 (PMC12333613; doi:10.1021/acs.jafc.5c04345)
Supplement: Supplementary file 1 [file jf5c04345_si_001.pdf]

Supplementary Information

Identification of slime mold metabolites that confer protection to commercial crops against root-knot nematodes

Kana Y. Hayashi [a], Yukiko Nagamatsu [b], Moemi Kawano [a], Sayaka Fuchimoto [a]

Tsuyoshi Araki [c], and Tamao Saito [c*]

[a] Graduate School of Science and Technology, Sophia University, Chiyoda-ku, Tokyo 102-8554, Japan

[b] Environmental Science Research Institute, Panefri Industrial Co., Ltd. , Naha, Okinawa 903-0815, Japan

[c] Faculty of Science and Technology, Sophia University, Chiyoda-ku, Tokyo 102-8554, Japan

4 Supplemental Figures

2 Supplemental Tables

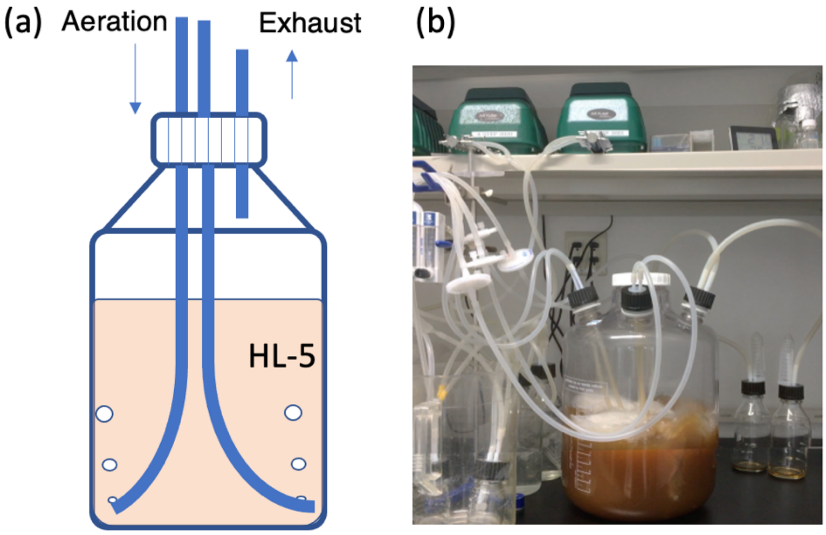

Figure S1 Large scale culture with the tank and preparation of CM

(a) Schematic diagram of a tank for cultivation. (b) Photograph of culture tank. KAx3 cells were cultured in a liquid medium HL-5. A system was established for culturing in a 12-L tank containing 10 L of medium. The 12-L tank was vented using three tubes. The ventilation rate was gradually increased to 40 L/min on the third day, resulting in an increased cell density of 2–3 x 10^7 cells/mL. Late-growth or stationary phase cells were washed with sterile water or sterile KK_2-phosphate buffer (pH 6.2) and resuspended at a concentration of 1 x 10^8 cells/mL. The cells were then cultured statically in water or buffer at room temperature (22°C) for three days. The cells and supernatant were collected as CM.

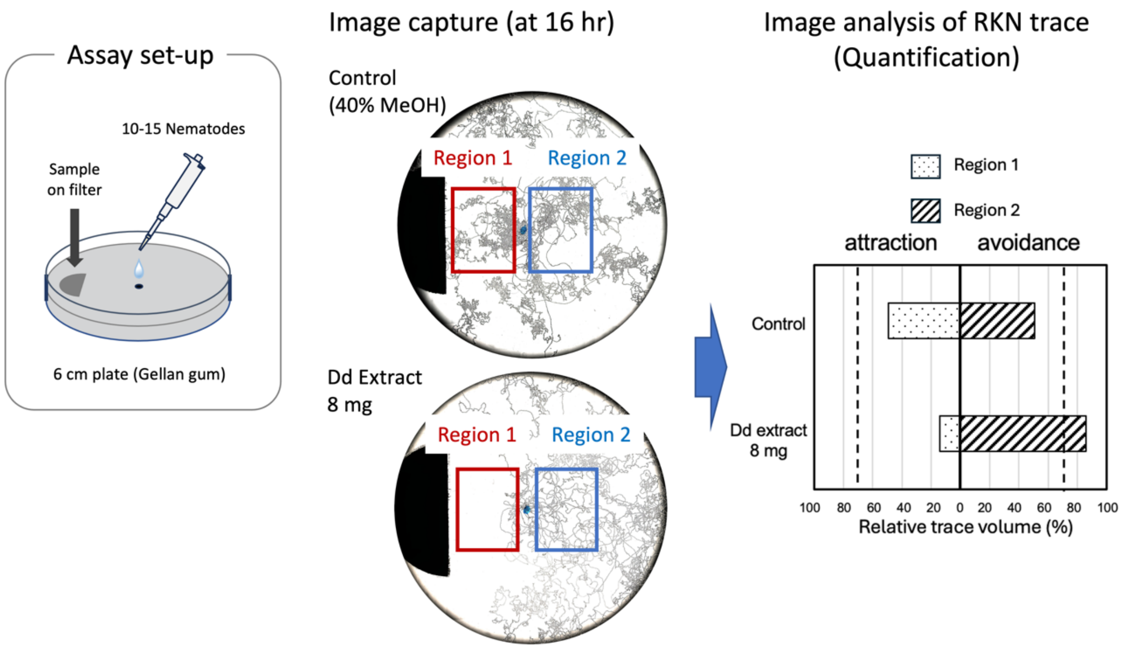

Figure S2 Schematic diagram of *in vitro* chemotaxis assay

A 6-cm diameter Petri dish containing 6 mL of KK_2 phosphate-buffered 1.5% gellan gum served as a tester plate, onto which dried filter paper saturated with the extract or samples and dried was placed. Approximately 10–15 juvenile nematodes were placed at the center of a Petri dish 1.2 cm away from the filter paper and allowed to migrate for 16 h. Snapshots of their movement were obtained by high-contrast imaging of the juvenile trails captured using a microscope (AZ100, Nikon, Japan) equipped with a camera (FR-400C, Flovel, Germany). Raw images of the nematode trials were analyzed using open-source ImageJ software. Trace in Region 1 indicates attraction and trace in Region 2 indicates repulsion. The trace of nematodes in each area was converted into the number of pixels, and the behavior was quantified. If more than 70% of total trace was in Region 2, this was considered as an indicator for repulsion. In the *in vitro* chemotaxis assay, after repeating the experiment more than 100 times, no values exceeding 70% were observed in either region 1 or 2 in the 40% methanol control. This suggests that random movement does not produce values exceeding 70%. Although all experimental results were statistically analyzed, for screening purposes, where a large number of samples are analyzed, values exceeding 70% in either region were considered repellent or attractive, and this criterion was used.

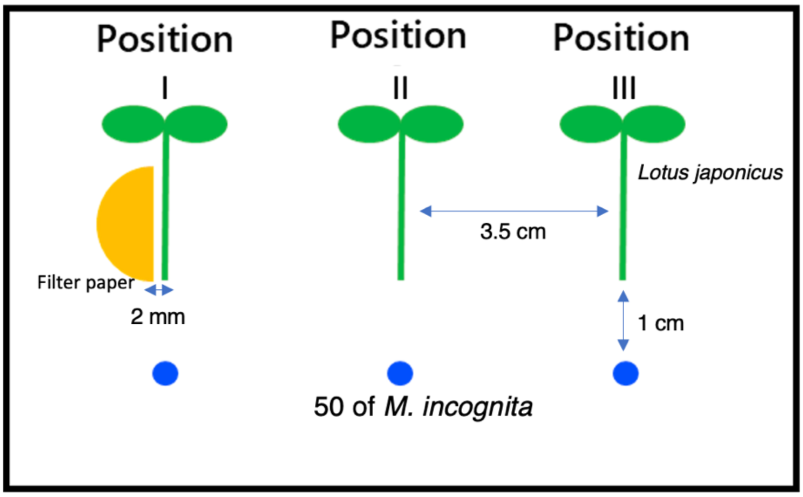

Figure S3 Schematic diagram of *in vitro* plant infection assay

Three seedlings of *Lotus japonicus* were placed on Lotus Broughton & Dilworth (B&D) agar medium prepared in rectangular Petri dishes at intervals of 3.5 cm. Filter paper containing CM or a repellent was placed 2 mm away from the root of Position I plant. Plants were placed at three positions (positions I to III) to investigate the area where compounds adsorbed on filter paper showed inhibitory effects on nematode infection.

Fifty larvae of *M. incognita* were placed 1 cm away from the tips of the roots of each plant. The dishes were dried, covered with lids, and incubated in a plant culture chamber for two days. After two days of incubation in the plant chamber, the number of infected nematodes was counted under a microscope (SMZ 745T, Nikon, Japan) following acid fuchsin staining (Bybd *et al.*).

40% methanol and 5 mg CM were used as negative and positive controls, respectively.

Bybd, D. W., Kirkpatrick, T., Barker, K. R. An improved technique for clearing and staining plant tissues for detection of nematodes (1983) J Nematol. 15 142-3

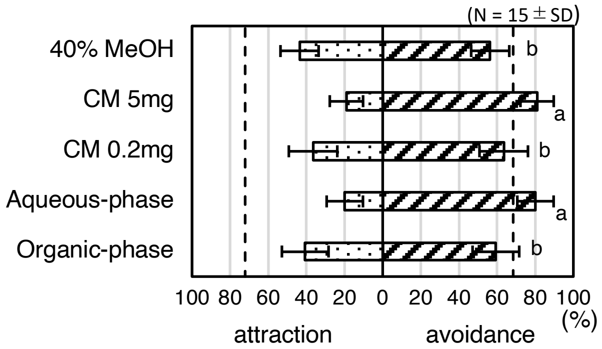

Figure S4 Repellent activity of Water-soluble repellent compounds

5 mg of CM was subjected to Bligh–Dyer separation, revealing that the repellent activity resided in the water rather than the organic phase. The comparison of 5 mg CM and a water layer of 5 mg CM showed no significant difference.

Values are expressed as mean ± SD of N = 15. Different letters denote significant differences between groups at $p < 0.05$.

Table S1 One month Wagener pot experiments

Table S1 shows the results of 10 experiments conducted over a period of one month (34–46 days).

First five experiments were conducted with a treatment frequency of three times CM irrigations per week.

In four out of the five experiments, the pest control value exceeded 50%. Trials 1 to 4 show these results. While Trial 1 achieved a control efficacy of 54.5%, a re-experiment under the same conditions yielded a control efficacy of 42.9% (not shown in the table), leading to the conclusion that the conditions for Trial 1 were inappropriate. In these four experiments, we initially used 200 mL of the solution, but later found that 150 mL was sufficient. Furthermore, when 2.4 g of CM was applied three times a week per plant, the pest control value was 50% or higher.

To reduce the amount of CM used per application, we conducted six experiments with seven times per week treatments (i.e., daily treatments). One of these trials achieved a pest control value of 41.7% (not shown in the table). The cause is unknown, but in this trial, plant growth was poor. The results are shown in Trials 5 to 8. From the series of results, we found that using 1.2 g of CM per plant per application consistently achieves a pest control value exceeding 50%.

Since pot tests use many nematodes, the amount of nematodes used varies. We prioritized using healthy nematodes over the number of nematodes and increasing the number of trials.

trial 1	sample	Number of times processed per week	Weight of dry CM and amount of water used per plant in a single treatment (g/mL)	Above-ground weight after application (g)	Average wright (g)	Root-knot index	Average of Root-knot index	Pest control value	Application period (number of inoculated nematodes/pot)
	control 1－1	3/week	0 g/200 mL	251		2		-	
	control 1－2	3/week	0 g/200 mL	217		3		-	
	control 1－3	3/week	0 g/200 mL	226	228.4	2	2.2	-	
	control 1－4	3/week	0 g/200 mL	266		2		-	
	control 1－5	3/week	0 g/200 mL	182		2		-	42 days (not recorded)
	CM1-1	3/week	1.3 g/ 200mL	300		1			
	CM1-2	3/week	1.3 g/200 mL	362		1			
	CM1-3	3/week	1.3 g/ 200mL	257	289	1	1	54.5	
	CM1-4	3/week	1.3 g/ 200mL	267		1			
	CM1-5	3/week	1.3 g/ 200mL	259		1			

trial 2	sample	Number of times processed per week	Weight of dry CM and amount of water used per plant in a single treatment (g/mL)	Above-ground weight after application (g)	Average wright (g)	Root-knot index	Average of Root-knot index	Pest control value	Application period (number of inoculated nematodes/pot)
	control 2-1	3/week	0 g/200 mL	214		3		-	
	control 2-2	3/week	0 g/200 mL	200	222.3	2	2.3	-	
	control 2-3	3/week	0 g/200 mL	253		2		-	41 days (5280 nematodes/pot)
	CM2-1	3/week	3.2 g/ 200mL	283		1			
	CM2-2	3/week	3.2 g/ 200mL	265	288.3	1	1	57.1	
	CM2-3	3/week	3.2 g/ 200mL	317		1			

trial3	sample	Number of times processed per week	Weight of dry CM and amount of water used per plant in a single treatment (g/mL)	Above-ground weight after application (g)	Average wright (g)	Root-knot index	Average of Root-knot index	Pest control value	Application period (number of inoculated nematodes/pot)
	control 3-1	3/week	0 g/150mL	178	184	3	3	-	34 days (4800 nematodes/pot)
	control 3-2	3/week	0 g/150mL	190		3		-	
	control 3-3	3/week	0 g/150mL	160		3		-	
	control 3-4	3/week	0 g/150mL	208		3		-	
	CM3-1	3/week	2.4 g/ 150 mL	231	223	1	1.5	50	
	CM3-2	3/week	2.4 g/ 150 mL	222		1			
	CM3-3	3/week	2.4 g/ 150 mL	230		2			
	CM3-4	3/week	2.4 g/ 150 mL	209		2			

trial 4	sample	Number of times processed per week	Weight of dry CM and amount of water used per plant in a single treatment (g/mL)	Above-ground weight after application (g)	Average wright (g)	Root-knot index	Average of Root-knot index	Pest control value	Application period (number of inoculated nematodes/pot)
	control 4-1	3/week	0 g/150mL	169	193.8	1	2	-	42 days (5000 nematodes/pot)
	control 4-2	3/week	0 g/150mL	199		1		-	
	control 4-3	3/week	0 g/150mL	210		3		-	
	control 4-4	3/week	0 g/150mL	197		3		-	
	CM4-1	3/week	2.4 g/ 150 mL	189	205.5	1	1	50	
	CM4-2	3/week	2.4 g/ 150 mL	252		1			
	CM4-3	3/week	2.4 g/ 150 mL	195		1			
	CM4-4	3/week	2.4 g/ 150 mL	186		1			

trial 5	sample	Number of times processed per week	Weight of dry CM and amount of water used per plant in a single treatment (g/mL)	Above-ground weight after application (g)	Average wright (g)	Root-knot index	Average of Root-knot index	Pest control value	Application period (number of inoculated nematodes/pot)
	control 5-1	7/week	0 g/150mL			3	2.8	-	42 days (5000 nematodes/pot)
	control 5-2	7/week	0 g/150mL			3		-	
	control 5-3	7/week	0 g/150mL			3		-	
	control 5-4	7/week	0 g/150mL			2		-	
	CM5-1	7/week	2.4 g/ 150 mL			1	1	63.6	
	CM5-2	7/week	2.4 g/ 150 mL			1			
	CM5-3	7/week	2.4 g/ 150 mL			1			
	CM5-4	7/week	2.4 g/ 150 mL			1			

For trial 5, the weight of the sample after treatment is not recorded.

trial 6	sample	Number of times processed per week	Weight of dry CM and amount of water used per plant in a single treatment (g/mL)	Above-ground weight after application (g)	Average wright (g)	Root-knot index	Average of Root-knot index	Pest control value	Application period (number of inoculated nematodes/pot)
	control 6-1	7/week	0 g/150mL	183	175.5	3	3	-	46 days (3000 nematodes/pot)
	control 6-2	7/week	0 g/150mL	178		3		-	
	control 6-3	7/week	0 g/150mL	162		3		-	
	control 6-4	7/week	0 g/150mL	179		3		-	
	CM6-1	7/week	1.2 g/150 mL	336	334.5	1	1	66.7	
	CM6-2	7/week	1.2 g/150 mL	355		1			
	CM6-3	7/week	1.2 g/150 mL	315		1			
	CM6-4	7/week	1.2 g/150 mL	372		1			

trial 7	sample	Number of times processed per week	Weight of dry CM and amount of water used per plant in a single treatment (g/mL)	Above-ground weight after application (g)	Average wright (g)	Root-knot index	Average of Root-knot index	Pest control value	Application period (number of inoculated nematodes/pot)
	control 7-1	7/week	0 g/150mL	161	127	3	3	-	42 days (3000 nematodes/pot)
	control 7-2	7/week	0 g/150mL	159		3		-	
	control 7-3	7/week	0 g/150mL	10		3		-	
	control 7-4	7/week	0 g/150mL	178		3		-	
	CM7-1	7/week	1.2 g/150 mL	227	236	1	1	66.7	
	CM7-2	7/week	1.2 g/150 mL	227		1			
	CM7-3	7/week	1.2 g/150 mL	238		1			
	CM7-4	7/week	1.2 g/150 mL	251		1			

trial 8	sample	Number of times processed per week	Weight of dry CM and amount of water used per plant in a single treatment (g/mL)	Above-ground weight after application (g)	Average wright (g)	Root-knot index	Average of Root-knot index	Pest control value	Application period (number of inoculated nematodes/pot)
	control 8-1	7/week	0 g/150mL	130	134	3	2.8	-	41days (4400 nematodes/pot)
	control 8-2	7/week	0 g/150mL	137		2		-	
	control 8-3	7/week	0 g/150mL	135		3		-	
	control 8-4	7/week	0 g/150mL	134		3		-	
	CM8-1	7/week	1.2 g/150 mL	178	179.5	1	1.3	54.5	
	CM8-2	7/week	1.2 g/150 mL	180		1			
	CM8-3	7/week	1.2 g/150 mL	178		1			
	CM8-4	7/week	1.2 g/150 mL	182		2			

Table S2 Metabolomic analysis of CM

Metabolomic analysis of the water-soluble components of CM was performed by the Chemicals Evaluation and Research Institute in Japan. A 10 μL sample was extracted via the Bligh–Dyer method with a specified amount of internal standard (2-isopropylmalic acid), and the resulting aqueous phase was lyophilized. The lyophilized samples were derivatized using a 2-methoxyamine hydrochloride pyridine solution and N-methyl-N-trimethylsilyl trifluoroacetamide. Samples were analyzed using a gas chromatography/tandem mass spectrometer (GCMS-TQ8030 Shimadzu, Japan) equipped with a BPX-5 column (30 m, inner ϕ 0.25 mm; Trajan Scientific and Medical, Australia). Table shows identified 126 compounds and L-lysine.
Therefore, we tested the repellent activities of 127 compounds containing 20 proteinogenic amino acids. All compounds were commercially available.
Metabolomics analysis was performed seven times in total, and a total of 126 compounds were obtained. The content was determined based on the area ratio of each compound, and variations were observed in each analysis.

#1	1,5-Diaminopentane
#2	1,6-Anhydro-β-D-glucose
#3	2-Aminoadipic acid
#4	2-Aminobutyric Acid
#5	2-Aminoethanol
#6	2-Aminopimelic Acid
#7	2-Deoxy-glucose
#8	2-Deoxyuridine
#9	2-Hydroxybutyric acid
#10	2-Hydroxyglutaric acid
#11	2-Hydroxyisocaproic acid (DL-Leucic acid)
#12	2-Keto-isovaleric acid
#13	3-Aminoisobutyric Acid
#14	3-Aminopentanedioic acid (3-Aminoglutaric acid)
#15	3-Aminopropanoic acid (beta-Alanine)
#16	3-Hydroxybutyric acid
#17	3-Hydroxyisobutyric acid (3-HIBA)
#18	3-Hydroxyisovaleric acid
#19	3-Hydroxypropionic Acid
#20	3-Methyl-2-oxovaleric acid
#21	3-Phenylactic Acid
#22	3-Ureidopropionic Acid
#23	4-Hydroxybenzoic acid
#24	4-Hydroxyphenylacetic acid
#25	4-Hydroxyphenyllactic acid (4HPLA)
#26	4-Hydroxyphenylpyruvic acid
#27	4-Hydroxyproline
#28	4-Methyl-2-oxopentanoic acid (2-Ketoisocaproic acid)
#29	5-Aminovaleric acid
#30	5-Methoxytryptamine
#31	7-Methylguanine
#32	Adenine
#33	Alanine
#34	Arabitol
#35	Arginine
#36	Ascorbic acid
#37	Asparagine
#38	Aspartic acid
#39	Benzoic acid
#40	Citric acid
#41	Cysteine
#42	Cytosine
#43	Dihydroxyacetone phosphate
#44	Elaidic acid
#45	Erythritol
#46	Fructose
#47	Fumaric acid
#48	Galactose
#49	Gluconic acid
#50	Glucosamine
#51	Glucose
#52	Glutamic acid
#53	Glutamine
#54	Glutaric acid
#55	Glyceric acid
#56	Glycerol
#57	Glycerol 2-phosphate
#58	Glycerophosphoric acid (Glycerol-3-phosphate)
#59	Glycine
#60	Glycolic acid
#61	Guanine
#62	Guanosine
#63	Heptadecanoic acid (Margaric acid)
#64	Hippuric acid
#65	Histidine
#66	Hypoxanthine
#67	Inosine
#68	Isoleucine
#69	Isomaltose
#70	Kynurenine
#71	L-Pyroglutamic acid (5-Oxoproline)
#72	Lactic acid/
#73	Lactitol Monohydrate
#74	Leucine
#75	Lyxose
#76	Maltose
#77	Mannitol
#78	Mannose
#79	Methionine
#80	myo-Inositol
#81	Nicotinic acid
#82	Norepinephrine
#83	Nα-Acetyl-L-glutamine
#84	O-Acetylserine
#85	Oleic acid
#86	Ornithine
#87	Palmitoleic acid (cis-9-Hexadecenoic Acid)
#88	Pantothenate
#89	Phenylacetic acid
#90	Phenylalanine
#91	Phenylpyruvic acid
#92	Phosphoric acid
#93	Proline
#94	Psicose
#95	Putescine (1,4-Butanediamine)
#96	Pyridoxine
#97	Pyruvic acid
#98	Rhamnose
#99	Ribitol
#100	Ribose
#101	Ribulose
#102	Serine
#103	Sorbose
#104	Spermidine
#105	Succinic acid
#106	Sucrose
#107	Tagatose
#108	Threitol
#109	Threonine
#110	Thymidine
#111	Thymine
#112	Trehalose
#113	Tryptamine
#114	Tryptophan
#115	Tyramine
#116	Tyrosine
#117	Uracil
#118	Urea
#119	Uridine
#120	Urocanic acid
#121	Valine
#122	Xantine
#123	Xylitol
#124	Xylose
#125	Xylulose
#126	γ-Aminobutyric acid (GABA)
#127	Lysine
